# The relationship between self-esteem and mobile phone addiction among college students: The chain mediating effects of social avoidance and peer relationships

**DOI:** 10.3389/fpsyg.2023.1137220

**Published:** 2023-04-13

**Authors:** Chunmei Chen, Yuanyi Shen, Shuai Lv, Bo Wang, Yujie Zhu

**Affiliations:** ^1^Teachers College, Jimei University, Xiamen, Fujian, China; ^2^School of Aerospace Engineering, Xiamen University, Xiamen, Fujian, China; ^3^Institute of Education, Xiamen University, Xiamen, Fujian, China; ^4^Office of Development Planning, Shenzhen Polytechnic, Shenzhen, Guangdong, China; ^5^School of Marine Culture and Law, Jimei University, Xiamen, Fujian, China

**Keywords:** self-esteem, mobile phone addiction, social avoidance, peer relationship, chain mediation, chain mediating effects

## Abstract

**Introduction:**

Mobile phone addiction has a negative impact on the physical and mental health of college students, which has attracted extensive attention from scholars.

**Methods:**

In this study, we investigated the mechanism of the influence of self-esteem on mobile phone addiction among 694 college students using the Self-Esteem Scale, the Mobile Phone. Addiction Scale, the Peer Relationship Scale and the Social Avoidance and Distress Scale.

**Results:**

The results showed that (1) self-esteem significantly and negatively predicted mobile phone addiction; (2) self-esteem influenced mobile phone addiction through the mediating effect of social avoidance; (3) self-esteem influenced mobile phone addiction through the mediating effect of peer relationships; and (4) social avoidance and peer relationships played a chain mediating role in the influence of self-esteem on mobile phone addiction.

**Discussion:**

These findings can help researchers and educators better understand the underlying mechanisms of the relationship between self-esteem and mobile phone addiction and to provide practical and effective operational suggestions for the prevention and intervention of mobile phone addiction among college students.

## 1. Introduction

On August 31, 2022, “The 50th China Statistical Report on Internet Development” released by China Internet Network Information Center (CINIC) (2022) showed that as of June 2022, the number of Chinese Internet users was 1.051 billion, of which the number of mobile phone users reached 1.047 billion, an increase of 17.85 million compared with December 2021. The proportion of Internet users using mobile phones to access the Internet was 99.6%, basically the same as in December 2021. College students are an important group of mobile phone users. The 5th generation mobile communication technology (5G) is a new generation broadband mobile communication technology with the characteristics of high speed, low delay and large connection. Its quick development has made mobile phones more powerful. However, the excessive and unreasonable use of mobile phones by college students has brought many negative effects on their physical and mental health, resulting in the emergence of mobile phone addiction. Li and Ren (2018) argue that mobile phone addiction is essentially a behavioral addiction that arises during a person’s interaction with a mobile phone and is a manifestation of inappropriate mobile phone use. Griffiths (1996) regards it as a human–computer interaction and non-chemical behavioral addiction, which is also a technological addiction. Lapierre et al. (2019) consider mobile phone addiction as an uncontrollable and excessive use of mobile phones that adversely affects an individual’s daily life. Billieux (2012) makes a similar point to Lapierre et al. He also lists four pathways that lead to this behavior, including the impulsive pathway, the relationship maintenance pathway, the extra-personal disposition pathway, and the cyber addiction pathway that acts indirectly. Many researchers point out that mobile phone addiction will manifest itself through a series of symptoms, such as mood modification, conflict, relapse, withdrawal and so on (Griffiths, 2005; Yen et al., 2009). This addiction can lead to out-of-control behaviors that cannot be stopped despite the negative consequences (Carnes, 1983; Billieux et al., 2008). Most addicts are aware that mobile phone addiction is harmful to their physical and mental health, but they still cannot control themselves to reduce their mobile phone use. Elhai et al. (2017) state that mobile phone addiction has a certain impact on the physical and mental health of college students. The physiological, psychological and social functioning of the individual is significantly impaired (Jiang and Zhao, 2016; Hu, 2023). It would cause physiological reactions such as tingling of the hands and feet, palpitations, dizziness (Yen et al., 2009; Thomée et al., 2011), depression, anxiety and disruption of sleep (Gilbert et al., 2015; Alhassan et al., 2018; Saied et al., 2022), and reduce cognitive flexibility (Derks and Bakker, 2012; İnal and Serel Arslan, 2021). Ding et al. (2022a) also find that smartphone addition was positively correlated with alexithymia, attachment anxiety, negative emotions and attachment avoidance. There is a high positive correlation between alexithymia and mobile phone addiction among mainland Chinese students (Huang et al., 2022). In addition, mobile phone addiction increases inert thinking (Derks and Bakker, 2012) and reduces teenagers’ social skills (Blöte et al., 2015; Lepp et al., 2016), even leads to interpersonal difficulties (Lepp et al., 2014). In conclusion, mobile phone addiction has become a major problem for college students and has attracted widespread attention from scholars. To this end, the aim of this study was to investigate the mechanisms influencing mobile phone addiction among college students.

### 1.1. The relationship between self-esteem and mobile phone addiction

Marlatt et al. (1988) notes that self-esteem has been associated with addictive behaviors. Competency-based and value-based or “virtue” are seen as two basic sources of self-esteem (Brissett, 1972; Brown, 1998). Rosenberg (1979) regards self-esteem as one’s overall sense of worthiness to be a person. Coopersmith (1989) argues that self-esteem is a relatively stable evaluation of oneself that one makes and maintains, often as a judgment of self-worth. Swann (1996) makes a similar point when he suggests that self-esteem is closely related to our perceptions of who we are. Liu and Wang (2017) state that self-esteem is a complex structure of individuals’ emotions, thoughts, and behaviors connected to each other, and is a protective mental health factor that influences the way individuals perceive and behave. Empirical studies have been conducted to show that self-esteem has a negative impact on mobile phone addiction. Low self-esteem is more likely to develop mobile phone addiction (Khang et al., 2013; Park and Lee, 2014; You et al., 2019; Ding et al., 2022b). High self-esteem may be a protective factor against mobile phone addiction (Wang et al., 2017). Self-esteem significantly predicts the phenomenon of cell phone addiction among adolescents. Adolescents with low self-esteem are more likely to vent their emotions and release stress through the Internet for immediate psychological satisfaction, which leads them to frequent cell phone use (Lannoy et al., 2020; Kong et al., 2022). In summary, hypothesis H1 is proposed.

*H1*: There is a significant effect of self-esteem on mobile phone addiction.

### 1.2. The mediating effect of social avoidance

According to social motivation theory, social avoidance is a subtype of social withdrawal (Coplan et al., 2015). Watson and Friend (1969) point out that social avoidance refers to the internal psychological experience and external behavioral manifestations of individuals who avoid interaction and fear rejection in social situations. It includes the fear of negative evaluation, the emotional experience and psychological feelings of shyness, nervousness, and avoidance in social situations, and the defensive tendency of individuals to speak little and not express their true opinions in social groups. When individuals experience negative experiences in social activities for a long time, they will unconsciously avoid the behavior of interacting with others, which eventually leads to the decline of self-esteem and the degradation of interpersonal skills (Beyens et al., 2016). Hong (2011) notes that there is a remarkable negative correlation between self-esteem and social avoidance and social anxiety. In addition, self-esteem plays an important role in the prediction of social avoidance and social anxiety. Yuan et al. argue that low self-esteem levels of social avoidance are more susceptible to interpersonal trust issues, which cause feelings of social anxiety (Yuan et al., 2022). It is evident that self-esteem is associated with social avoidance and social anxiety. In addition, some studies have shown that social avoidance among college students correlates with the presence of Internet addiction. Mobile phones may be addictive because they may be used to avoid situations that are distasteful to the user (Bianchi and Phillips, 2005). Mobile phone use can reduce individuals’ socially relevant threat and anxiety. Individuals are more likely to use their phones as a substitute for social contact (Lepp et al., 2014). Yücens and Üzer (2018) note that there is a strong correlation between social avoidance and internet addiction. Social anxiety plays a role in internet addiction. Gao et al. (2021) point out that people with social avoidance disorder habitually use online communication to fulfill their real communication needs and minimize the frequency of real interpersonal interactions as much as possible. For this reason, social avoidance may play a mediating moderating role. In summary, hypothesis H2 is proposed.

*H2*: Social avoidance mediates the relationship between self-esteem and mobile phone addiction.

### 1.3. Mediating effects of peer relationships

Peer relationship is an important part of interpersonal relationships, which refers to interpersonal relationships formed and developed during the interaction between people of similar age or psychological development level. It mainly includes two aspects of popularity and depth of friendship (Breines and Ayduk, 2015). Young people’s self-esteem is related to peer relationships (Rosenberg, 1965), especially if their self-worth is related to the approval of others (Crocker and Wolfe, 2001). Birkeland et al. (2014) state that peer acceptance has a protective effect on adolescent self-esteem. Social connection theory suggests that low self-esteem weakens an individual’s connection to society, causing individuals to have fewer social connections, which in turn affects peer relationships (Xin et al., 2007). High self-esteem enhances individuals’ interpersonal confidence and enables individuals to live in harmony with others and have good peer relationships (Zhang, 2014). A number of studies have shown a significant positive correlation between peer relationships and adolescent self-esteem (McLean and Jennings, 2012; Vanhalst et al., 2014; Thompson et al., 2016). Good peer relationships can bring positive emotional feedback to adolescents, thereby increasing self-esteem levels (Mann and Blumberg, 2022). In addition, it has been established that peer victimization is a significant risk factor for adolescent Internet addiction (Jia et al., 2018). Improving the quality of peer relationships is an effective way to prevent and quality Internet addiction (Reiner et al., 2017). Therefore, peer relationship may be a mediating variable between self-esteem and mobile phone addiction. In summary, hypothesis H3 is proposed.

*H3*: Peer relationships mediate the relationship between self-esteem and mobile phone addiction.

### 1.4. Chain mediation effects of social avoidance and peer relationships

Tu et al. (2022) put forward that higher levels of social avoidance were associated with lower levels of peer relations and lower levels of social avoidance are associated with higher levels of peer relations. Many scholars have studied peer rejection, which is a negative form of peer relations (Williams et al., 2000). Children’s levels of social avoidance positively predict levels of peer rejection (Bowker and Raja, 2011; Bowker et al., 2012). Coplan et al. (2016) screen socially avoidant children with the Shyness Scale and the Solitary Preference Scale, finding that socially avoidant children had higher levels of peer rejection and peer bullying than the average child. Molden et al. (2009) suggest that individuals who are rejected will develop a sense of loss, leading to a more prevention-oriented response and distance from social contact. Social avoidance displays such as shyness and unsociability significantly predict low levels of peer liking and high levels of peer rejection (Eggun et al., 2022). In summary, there is an influential relationship between social avoidance and peer relationships. In addition, some scholars have also studied the relationship between social media and social avoidance. Paz et al. (2017) argue that individuals with low self-esteem experience high levels of distress in interpersonal relationships in a number of important areas. The use of social media can effectively help individuals reduce the distressing experience due to suffering from social exclusion and alleviate the negative effects (Lin et al., 2017). Baumeister and Tice (1990) state that peer exclusion can lead to unmet needs for belonging and relationships, causing individuals to experience avoidance of real social situations, which is an important risk factor for exacerbating problematic social media use among adolescents. Li (2020) studies 965 Guangzhou (*N* = 747) and Macau (*N* = 216) adolescents. He found that the mediating effect of social avoidance between peer rejection and problematic social media use among adolescents in Guangzhou was established. To this end, hypothesis H4 is formulated.

*H4*: Social avoidance and peer relationships play a chain mediating role between self-esteem and mobile phone addiction.

This study constructed a chain mediation model to examine the influence of self-esteem on mobile phone addiction and the mediating role of social avoidance and peer relationships between the two in college students, in order to provide new ideas for mobile phone addiction prevention and intervention among college students.

## 2. Research methodology

### 2.1. Object of the study

A convenience sampling method was used to select college students from four universities in China for the questionnaire survey, and 694 valid questionnaires were collected after sorting. The subjects’ ages ranged from 17 to 23 years old (M = 20.16 years old, SD = 1.31), including 205 (29.6%) in the first year of college, 193 (27.8%) in the second year of college, 157 (22.6%) in the third year of college, and 139 (20.0%) in the fourth year of college.

### 2.2. Research tools

#### 2.2.1. Self-esteem scale

A Chinese version of The Self - Esteem Scale (SES) developed by Rosenberg was used (Wang et al., 1999). The scale is a 10-item scale with a 5-point scale in which 5 items are reverse scored and all items are summed. The higher the total score, the higher the level of self-esteem of the individual. The Cronbach’s alpha coefficient for this questionnaire in this study was 0.76.

#### 2.2.2. Mobile phone addiction scale

The Mobile Phone Addiction Tendency Scale for college students designed by Xiong et al. (2012) to measure the tendency of mobile phone addiction among college students, which consists of 16 items in four areas: withdrawal symptoms, emergent behaviors, social soothing and mood changes. The Cronbach’s alpha coefficient of this questionnaire in this study was 0.93.

#### 2.2.3. Peer relationship scale

The peer relationship scale developed by Asher and revised by Zhang (2008), a Chinese scholar, consists of 16 items in three areas: welcome, rejection, and loneliness, with a 5-point scale. The Cronbach’s alpha coefficient of this questionnaire in this study was 0.93.

#### 2.2.4. Social avoidance and distress scale

The Social Avoidance and Distress Scale (SAD) developed by Watson and Friend, revised by Peng (Wang et al., 1999), was used, containing 28 questions, 14 of which were used to measure social avoidance. The higher the total score, the higher the level of social avoidance of the individual. The Cronbach’s alpha coefficient of this questionnaire in this study was 0.851.

### 2.3. Research procedures

SPSS 26.0 was used for descriptive statistics and Pearson correlation analysis. To ensure the accuracy of the results, the variance inflation factor (VIF) method was used for covariance testing (if VIF > 10,it means that there is a serious covariance problem between the variables and the corresponding variables need to be excluded). Model 6 in the process plug-in prepared by Hayes (2017) was used for chain mediated effects analysis, and the significance of the mediated effects was tested using the bias-corrected percentile Bootstrap method. Statistical significance was considered if the 99% confidence interval did not contain a value of 0 (Erceg-Hurn and Mirosevich, 2008). In addition, prior to analyzing the data, a common method bias test was conducted using the Harman single factor test (Podsakoff et al., 2003).

## 3. Research results

### 3.1. Common method bias test

The issue of common method bias may arise when data are collected using the self-report method. The common method bias test was conducted using the Harman single factor test. The results showed that there were eight principal components with eigenvalues greater than one, and the first principal component explained 34% of the variance, which was below the critical criterion of 40%. Therefore, there is no serious common method bias in this study.

### 3.2. Descriptive statistics and correlation analysis of each variable

The means, standard deviations, and Pearson product difference correlation coefficients between the variables for self-esteem, mobile phone addiction, peer relationships, and social avoidance were given in Table 1. The correlations among the variables all reached the significance level, among which, all the correlations among the variables showed negative correlations, except for the two influential relationships of self-esteem and peer relationship, and mobile phone addiction and social avoidance, which suggested positive correlations.

**Table 1:** Descriptive statistics and correlation matrix for each variable.

	Mean	Standard deviation	Mobile phone addiction	Social avoidance	Self-esteem	Peer relationships
Mobile phone addiction	2.289	0.725	1			
Social avoidance	2.657	0.549	0.603**	1		
Self-esteem	3.425	0.572	−0.523**	−0.229**	1	
Peer relationships	3.908	0.682	−0.659**	−0.420**	0.673**	1

*p < 0.05; **p < 0.01.

### 3.3. The relationship between self-esteem and mobile phone addiction: A chain mediation model

The above analysis indicated significant correlations among the variables and possible covariance. Therefore, the predictor variables in the equation were standardized and covariance diagnosed before testing for effects. The results showed that the variance inflation factors (1.375, 1.277, and 1.210) for all predictor variables were less than 5. Therefore, the data used in this study did not have serious co-integration problems and were suitable for further testing of mediation effects. The process plug-in developed by Hayes was used to assess the 95% confidence interval (CI) of the mediating effect of social avoidance and peer relationship in the effect of self-esteem on mobile phone addiction (bootstrap sample size of 5,000), and the results of the chain mediation model were developed as shown in Table 2.

**Table 2:** The regression equation of chain mediation.

Regression equation (*N* = 694)		Fitting index			Coefficient and significance	
Outcome variables	Predictor variables	R	$R^{2}$	F	β	t
Social avoidance	Constants	0.229	0.052	38.342	47.752***	27.642
	Self-esteem				−0.308***	−6.191
Peer relationships	Constants	0.726	0.528	385.833	37.542***	14.933
	Self-esteem				1.162***	22.665
	Social avoidance				−0.398***	−10.454
Mobile phone addiction	Constants	0.763	0.583	320.886	50.772***	17.553
	Self-esteem				−0.377***	−5.561
	Social avoidance				0.616***	14.992
	Peer relationships				−0.386	−10.152

***p < 0.001.

**Figure 1:**
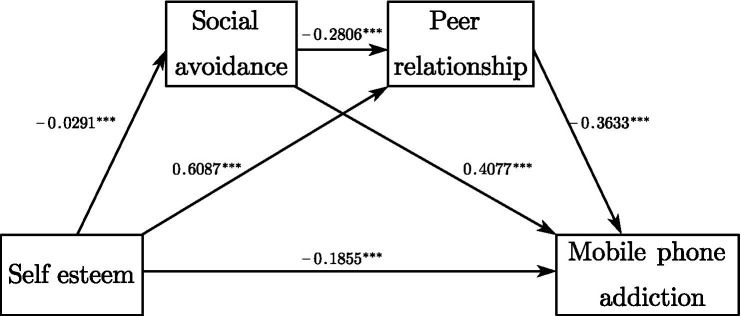
The chain mediation model. ***p < 0.001.

The results showed that self-esteem positively and significantly predicted peer relations (β = 0.609, *p* < 0.001) and negatively and significantly predicted social avoidance and mobile phone addiction (β = −0.029, p < 0.001; β = −0.186, p < 0.001). Hypothesis H1 was supported. Social avoidance positively predicted mobile phone addiction (β = 0.408, p < 0.001) and negatively predicted peer relations (β = −0.281, *p* < 0.001). Peer relations significantly and negatively predicted mobile phone addiction (β = −0.363, *p* < 0.001) (see Figure 1).

Further examination of the mediating effects (see Table 3) revealed that the Bootstrap 95% CI for the total indirect effect of social avoidance and peer relationships in the effect of self-esteem on mobile phone addiction was (−0.806, −0.570), excluding 0. This indicated that social avoidance and peer relationships were mediating variables in the effect of self-esteem on mobile phone addiction. The total indirect effect of both was −0.686, accounting for a total effect of 64.55%. This mediating effect was composed of three main pathways.

**Table 3:** Bootstrap analysis of the mediation effect test.

	Effect	Boot SE	Boot LL CI	Boot UL CI	Relative mediation effect
Total Indirect effect	−0.686	0.060	−0.806	−0.570	64.55%
Indirect effect 1	−0.190	0.032	−0.255	−0.128	17.84%
Indirect effect 2	−0.449	0.053	−0.557	−0.349	42.25%
Indirect effect 3	−0.047	0.010	−0.068	−0.030	4.46%

Indirect effect 1: Self-esteem → Social → Mobile phone.

Indirect effect 2: Self-esteem → peer → Mobile phone.

Indirect effect 3: Self-esteem → Socialization → Companion → Mobile phone.

1. Self-esteem → social avoidance → mobile phone addiction [95% CI = (−0.255, −0.128), standard error (SE) = 0.032], with a mediating effect of −0.190, accounting for 17.84% of the total effect. Hypothesis 2 was supported.
2. Self-esteem → peer → mobile phone [95% CI = (−0.557, −0.348), SE = 0.053], with a mediating effect of −0.449, accounting for 42.25% of the total effect. Hypothesis 3 was supported.
3. Self-esteem → socialization → peer → mobile phone [95% CI = (−0.068, −0.030), SE = 0.010], with a mediating effect of −0.047, accounting for 4.46% of the total effect. Hypothesis 4 was supported.

## 4. Discussion and conclusions

This study explored the effect of self-esteem on mobile phone addiction and the chain mediating effect of social avoidance and peer relationships in it. The study showed that self-esteem is negatively associated with mobile phone addiction. The higher the individual’s self-esteem, the lower the degree of mobile phone addiction. Social avoidance and peer relationships partially mediate the effect between self-esteem and mobile phone addiction. In addition, social avoidance and peer relationship effects are closely related, and both have a chain mediating effect in the process of self-esteem influencing mobile phone addiction.

Firstly, the results of this study noted that self-esteem negatively predicted mobile phone addiction, i.e., college students with higher self-esteem would have less mobile phone addiction, and conversely, college students with low self-esteem would have more mobile phone addiction. This is consistent with the findings of existing studies (Bianchi and Phillips, 2005; Li, 2016). Davis (2001) suggests that low self-esteem is a risk factor for Internet addiction. People with high self-esteem are more socially accepted, whereas people with low self-esteem have less social needs to meet and therefore desire more acceptance from others (Rudich and Vallacher, 1999). Therefore, people with low self-esteem may be more likely to develop social anxiety. Kocovski and Endler (2000) suggest that low self-esteem plays a key role in the development of social anxiety in college students (Kocovski and Endler, 2000). Increasing self-esteem may help people reduce social anxiety (Cheng et al., 2015; Ran et al., 2018). Ge et al. (2023) state that anxiety decreases the cognitive resources available to individuals for executive functioning, and impaired executive functioning can lead to individuals’ inability to control their behavior, which can lead to mobile phone addiction. Higher levels of social anxiety means that they are less able to experience pleasure in their daily lives (Wacks and Weinstein, 2021). According to Lannoy et al. (2020), people with high social anxiety tend to rely on the online world in their cell phones for emotional support, as a way to fill the gap in real interpersonal interactions and the need to gain a sense of identity and belonging. Studies have confirmed that the fear of missing out (FoMO) is closely related to mobile phone addiction (Sun et al., 2022; Zhang and Zhang, 2023). The more individuals are afraid of missing out on information, the higher their level of mobile phone addiction. College students with low self-esteem may be more inclined to reduce social anxiety through mobile phone use and therefore more prone to mobile phone addiction.

Secondly, the results of this study showed that social avoidance partially mediates the relationship between self-esteem and mobile phone addiction. That is, the higher the self-esteem of college students, the lower the level of social avoidance, and thus the lower the level of mobile phone addiction. This is consistent with Wu’s (2014) suggestion that self-esteem is significantly and negatively related to social avoidance. Cattell et al. (1961) suggests that individuals tend to choose avoidance behaviors when their self-image is questioned by the external environment. Watson and Friend (1969) argue that individuals who experience negative experiences of discomfort, maladaptive and stress in interpersonal interactions for long periods of time, or even fear of negative evaluation, may develop behaviors that lead them away from social groups. Edwards et al. (2022) note that self-esteem is a core component of the self. Individuals with high self-esteem have a clear perception of their self-concept, which helps them to export and express their ideas more comfortably in group activities and to be more easily accepted by the group, gaining satisfaction and self-confidence. Individuals with low self-esteem are more likely to experience anxious feelings of rejection during social activities due to excessive caution and fear, which because of their inherent lack of confidence and high sensitivity (Wang and Lei, 2021). College students with low self-esteem are more likely to have anxiety due to fear of external questioning and evaluation, and thus choose to avoid social behavior. Beidel et al. (2010) suggest that individuals with social anxiety are often afraid of social activities and are less likely to participate in them. They are accustomed to avoid social activities by being addicted to their mobile phones and seek identity and belonging in the online world as a way to reduce pain, anxiety and other negative emotions (Przepiorka et al., 2021). In addition, individuals who lack socialization are more likely to feel lonely. Loneliness avoidance may contribute to mobile phone addiction in young people (Ang et al., 2017; Li et al., 2021). College students with low self-esteem are more likely to develop social anxiety, avoid interpersonal interactions, and seek satisfaction through addiction to mobile phones.

Thirdly, the results of this study put forward that peer relationships partially mediate the relationship between self-esteem and mobile phone addiction. That is, the higher the self-esteem of college students, the better their peer relationships, and thus the lower their level of mobile phone addiction. Peer relationships have an important impact on the physical and mental health of college students. Gao et al. (2021) argue that positive peer relationships imply more emotional support, tangible companionship and mutual self-expression, which can effectively promote good school adjustment among adolescents and increase their sense of belonging at school, and thus reduces their dependence on cell phones. Liu et al. (2020) put forward that the better peer relationships college students have, the more likely they are to reap a sense of belonging and well-being in real life, and the less likely they are to be addicted to mobile phones. In addition, secure peer attachment may make individuals less prone to depression and anxiety symptoms (Gorrese, 2015). People with higher levels of attachment anxiety are more likely to use their mobile phone as a compensatory attachment target (Konok et al., 2016). Kim et al. (2017) consider that smartphone use is another option for individuals lacking secure attachment. Han et al. (2017) hold a similar view. They find a significant positive correlation between attachment anxiety and mobile phone dependence. Peer mutual exclusion and victimization often predicts more social anxiety (Su et al., 2016). The poorer the peer relationships of college students, the higher their level of attachment anxiety and the less likely they are to be addicted to mobile phones. Moreover, Gorrese and Ruggieri (2013) indicate that there is a significant correlation between peer attachment and self-esteem. The sense of security in peer attachment may be a protective factor for self-esteem fluctuations. The better the peer relationship, the higher the self-esteem of college students (Birkeland et al., 2014; Thompson et al., 2016). Good peer relationships can help college students adapt to new environments and balance role changes, i.e., good peer relationships predict higher levels of self-esteem among college students (Wright et al., 2020). College students with low self-esteem are more likely to have peer relationship problems because they are unable to receive care and support from their peers or even receive negative comments. For this reason, they are more likely to choose to indulge in mobile phones to gain emotional comfort.

Finally, this study found that social avoidance and peer relationship were closely related, and they constituted the middle link in the influence path of self-esteem → social avoidance → peer relationship → mobile phone addiction, and there was a chain mediating effect in the process of self-esteem influencing mobile phone addiction. That is, college students with stronger self-esteem have less tendency to social avoidance, and thus have better peer relationships and lower levels of mobile phone addiction. Social avoidance and peer relationships are closely related. Higher levels of social avoidance mean that individuals are in a more negative state in their peer relationships. Conversely, lower levels of socialization represent individuals acquiring more positive emotional experiences in peer relationships. This is similar to the findings of You et al. (2019). With the rapid development of the Internet, using mobile phones to access the Internet has become a very common daily behavior among college students. Salehan and Negahban (2013) find that the use of social net-working services (SNS) mobile applications is increasing and it is a significant predictor of mobile addiction. Mobile phone use has become a means for college students to maintain peer relationships and reduce social anxiety. The anonymity, timeliness, interactivity, and convenience of the Internet allow them to establish more connections with the outside world and thus avoid negative experiences that they may face with negative evaluations and anxiety-provoking situations. In addition, Gómez-Ortiz et al. (2017) suggest that there is a strong relationship between social anxiety and negative self-esteem. People who lack self-esteem and social skills, crave the approval of others and a sense of belonging are more likely to become addicted to their phones (Chen, 2018). In addition, Zwilling (2022) note that during the COVID-19 pandemic, college students’ use of cell phones for various purposes increased, i.e., constant access to digital screens for social contact, gaming entertainment, and other needs, exacerbating the emergence of cell phone addiction risk. This study confirmed that social avoidance and peer relationships have both partially mediated and chain-mediated effects in the process of self-esteem influencing mobile phone addiction.

## 5. Research value

### 5.1. Theoretical contribution

This study revealed the ways in which self-esteem, social avoidance, and peer relationships influence the phenomenon of mobile phone addiction among college students, and explored the chain mediating role of social avoidance and peer relationships. Most domestic and international studies have focused on the relationship between self-esteem and mobile phone addiction, but fewer studies have taken social avoidance and peer relationships as perspectives. This study explained the mechanism of self-esteem on the phenomenon of mobile phone addiction among college students from the perspectives of social avoidance and peer relationships, and emphasized the chain mediating effect of the two in it. To this end, this study can enrich the theoretical research related to the effects of social avoidance, peer relationships and self-esteem on mobile phone addiction. More and more people are aware of the harm caused by mobile phone addiction. In the general environment where the Internet is prevalent, college students’ mobile phone addiction deserves to raise more attention. There are many influencing factors that lead to mobile phone addiction among college students, among which social avoidance and peer relationship are two major aspects that cannot be ignored. The results of the study extend the theoretical construction of the mechanism of the occurrence of mobile phone addiction among college students, which can provide practical and effective empirical evidence for educators in colleges and universities to prevent and intervene in the phenomenon of mobile phone addiction, with certain guiding significance.

### 5.2. Research implications

#### 5.2.1. Improve college students’ self-esteem levels

According to Rosenberg (1965), self-esteem is a positive or negative attitude toward something specific to the self, which follows the law of judgment and the code of conduct of society. Self-esteem comes from acceptance and approval under certain social standards. Individuals with low self-esteem have lower acceptance and approval under certain social standards, hold negative attitudes toward self, have lower self-confidence, tend to avoid social behavior, and are not conducive to establishing good peer relationships. In such cases, they tend to seek other ways and means to enhance peer relationships, and the social features of mobile phones meet the needs of people with low self-esteem. However, over-reliance on mobile phones for peer communication and venting negative emotions may lead to mobile phone addiction. The results of this study further confirm that the tendency of mobile phone addiction can be effectively reduced by improving self-esteem. In view of this, universities should create more practical opportunities to develop students’ abilities in various areas and improve their competence so that they can gain a sense of accomplishment and being in demand. At the same time, universities can also instruct students to evaluate themselves objectively through classroom teaching and lectures to enhance their sense of self-esteem. Only when students are able to accept themselves and have confidence in themselves, they will be able to build relationships with others more positively and reduce their level of social anxiety and avoidance, thus reducing their use of mobile phones.

#### 5.2.2. Develop good peer relationships among college students

This study found that social avoidance can negatively affect their peer relationships. In a society that emphasizes group attachment and interpersonal harmony, groups that always actively avoid cultivating peer relationships are seen as selfish, rebellious or deviant, and thus rejected or bullied by their peers (Coplan et al., 2016). They are more likely to have the risk of peer relationship problems, and choose to seek spiritual solace in virtual ways such as mobile phones, resulting in mobile phone addiction. In contrast, groups with a higher level of acceptance and recognition and good peer relationships are less likely to have mobile phone addiction. Some college students are not good at establishing good relationships with their peers due to their personalities and habits. Especially some only children, who are used to various pampering from their families, may not know how to care, devote and maintain a good friendship. Although college students have reached adulthood, the role and spiritual intervention played by parents and teachers are especially important in their growth process. Parents and teachers should encourage college students to develop social relationships, form a sense of social belonging, care for others, and make their own contributions to the collective. Parents are supposed to do more to encourage college students to learn to express their inner thoughts, increase their active social awareness, and develop good social and behavioral habits. Universities should take the development of students’ interaction skills as an important teaching task and actively develop cooperative learning among students. For example, cooperative learning groups are supposed to be used as a basic form of interaction between dynamic elements of teaching and learning to promote the development of good peer relationships among students. Universities need to not only promote the development of students’ cognitive structures and abilities, but also promote the development of students’ “group” and “social” nature, and improve students’ socialization. The development of good peer relationships cannot be achieved without participation in activities. Colleges, parents and society need to form a joint effort to encourage college students to participate in social practice activities to enhance their immersive activity experience, which can improve students’ level of self-acceptance and recognition as well as foster good peer relationships, thus intervening in the formation of mobile phone addiction.

#### 5.2.3. Take care of the psychological growth of college students

Some college students may be in a state of high anxiety or even depression due to various reasons. Some other college students may be devastated after experiencing some big setbacks and lose their interest in people and things around them. If this negative state is not improved for a longer period of time, the students may become addicted to mobile phones and lose themselves. Colleges and universities should strengthen the mental health counseling of college students and provide professional counseling services to help this group of students get out of the downturn. The society should also create a free and harmonious atmosphere, so that college students can get timely help when they have psychological problems. Then they can build up self-confidence, improve self-esteem, be happy with themselves and tolerate others. College students themselves should strengthen physical exercise, get closer to nature and participate in more social practice activities after study, while reducing the time and frequency of mobile phone use. They should promote the healthy development of body and mind through more “natural contact” and “contact with people.”

### 5.3. Limitations and outlook

This study still has limitations. First, the sample was limited by the source of cross-sectional data, and remained inadequate in terms of confirmatory inferences about the causality of variables. Second, selection bias and potential threats may exist in the case of convenience sampling. Finally, the questionnaire was conducted without intervention due to time constraints. Therefore, as part of future research, follow-up studies should be designed and implemented using multiple data collection methods. Longitudinal studies should be conducted after the questionnaires have been administered. Future studies can use longitudinal data to verify the causal relationships of the variables of interest.

## Data availability statement

The raw data supporting the conclusions of this article will be made available by the authors, without undue reservation.

## Ethics statement

The studies involving human participants were reviewed and approved by the Ethics Committee of Jimei University. The patients/participants provided their written informed consent to participate in this study.

## Author contributions

CC designed the study and wrote the manuscript. YS and CC analyzed the data. SL collected the data. BW and YZ modified the manuscript. BW supervised the development of research and provided funding support. All authors contributed to the article and approved the submitted version.

## Funding

This study received funding from New Era Vocational Education Research Institute of China 2022 Annual Key Project (Project no. SZ22B05), 2022 Major Project of Educational Science Research of Shenzhen Polytechnic (Project no. 7022310045), and 2022 Guangdong Province Education Science Planning Project (Project No. 2022GXJK105) and Guangdong Social Science Planning Project in 2020 (Project no. GD20XJY48).

## Conflict of interest

The authors declare that the research was conducted in the absence of any commercial or financial relationships that could be construed as a potential conflict of interest.

## Publisher’s note

All claims expressed in this article are solely those of the authors and do not necessarily represent those of their affiliated organizations, or those of the publisher, the editors and the reviewers. Any product that may be evaluated in this article, or claim that may be made by its manufacturer, is not guaranteed or endorsed by the publisher.
